# Sharing Space: The Presence of Other Bodies Extends the Space Judged as Near

**DOI:** 10.1371/journal.pone.0114719

**Published:** 2014-12-10

**Authors:** Chiara Fini, Marcello Costantini, Giorgia Committeri

**Affiliations:** 1 Laboratory of Neuropsychology and Cognitive Neuroscience, Department of Neuroscience, Imaging and Clinical Sciences, University G. d′Annunzio, and ITAB, Foundation G. d′Annunzio, Chieti, Italy; 2 Mind, Brain Imaging and Neuroethics, Institute of Mental Health Research, University of Ottawa Ottawa, ON, Canada; University of Bologna, Italy

## Abstract

**Background:**

As social animals we share the space with other people. It is known that perceived extension of the peripersonal space (the reaching space) is affected by the implicit representation of our own and other's action potentialities. Our issue concerns whether the co-presence of a body in the scene influences our extrapersonal space (beyond reaching distance) categorization.

**Methodology/Principal Findings:**

We investigated, through 3D virtual scenes of a realistic environment, whether egocentric spatial categorization can be influenced by the presence of another human body (Exp. 1) and whether the effect is due to her action potentialities or simply to her human-like morphology (Exp. 2). Subjects were asked to judge the location ("Near" or "Far") of a target object located at different distances from their egocentric perspective. In Exp. 1, the judgment was given either in presence of a virtual avatar (Self-with-Other), or a non-corporeal object (Self-with-Object) or nothing (Self). In Exp. 2, the Self condition was replaced by a Self-with-Dummy condition, in which an inanimate body (a wooden dummy) was present. Mean Judgment Transition Thresholds (JTTs) were calculated for each subject in each experimental condition. Self-with-Other condition induced a significant extension of the space judged as “Near” as compared to both the Self-with-Object condition and the Self condition. Such extension was observed also in Exp. 2 in the Self-with-Dummy condition. Results suggest that the presence of others impacts on our perception of extrapersonal space. This effect holds also when the other is a human-like wooden dummy, suggesting that structural and morphological shapes resembling human bodies are sufficient conditions for the effect to occur.

**Conclusions:**

The observed extension of the portion of space judged as near could represent a wider portion of “accessible” space, thus an advantage in the struggle to survive in presence of other potential competing individuals.

## Introduction

Substantial evidence suggests that perception of the environment is shaped by our possibility to act, defined in terms of both body morphology [1]-[8] and energetic costs [9] (see [10] for a different view).

Regarding the space immediately around us it has been demonstrated that perception of affording features is enhanced when the affording object falls within the reachable space of the onlooker, thus allowing her to directly interact with it [11]. Also, distance judgments with respect to a given object have been shown to vary according to the action capabilities of the individual [6], [8], [12] and to the difficulty to pick up the object [3]. Hence, perceptual experience [11] and categorization [13] of the environment must be somehow constrained by our own motor system and his possibilities [14].

This is not the whole story though, indeed, we live in a world inhabited not only by inanimate things but also by other living and acting bodies. Previous studies have shown that our motor responsiveness is affected by the presence of another individual, able like-me to act in the reaching space. Specifically, the other's body is processed with our same action potentialities, indeed an object may afford a suitable motor act when is ready not only to our own hand but also to the other's hand [15]–[17]. In the same way, when we see an affordable object the other's action opportunities modulate our perception: observing someone reaching an object with a tool, makes us to perceive the object as closer [12], [18]. This evidence suggests that the human body is a visual stimulus which undergoes a special processing as a source of action, that could be remapped into our own.

In sum, our space perception and categorization are directly linked to what we can do in the surrounding peripersonal space and, importantly, to what also other bodies can do in their peripersonal space.

Regarding the space beyond reaching distance (i.e., extrapersonal space) [19]–[22], which we commonly share with other bodies, it seems to be tuned to the perceiver's opportunities to act as the peripersonal one. It appears to be categorized not only in relation with the relevant optical and ocular-motor variables, but also as a function of the costs associated with performing intended actions [4], [23], [24] and motivation to pursue a given goal [25]. We know, for instance, that wearing a heavy pack influences our distance judgments, making distances to appear greater [23]. There is also evidence that the apparent distance of a target within extrapersonal space is function of both its actual distance and the effort associated with intended actions directed to it [4]. Not only short-term factors such as fatigue, but also long-term factors, such as decline of the motor ability due to aging, influence the perceptual judgments. Older people, indeed, judge distances to be farther than younger adults [26] and interestingly, an actual reduction of the perceived capacity due to aging is not necessary to judge the distance as further, as young people primed with elderly categories, estimate the distances across a grassy field longer than their non-primed counterparts [27].

A relevant unexplored question is whether also the other's body, and not only our own, affects the perception of the extrapersonal space. To this regard, we have recently shown that target objects are judged as closer to a human agent rather than to a static object, a wooden dummy or a body without available movement potentialities [28]. In other words, when explicitly assuming another body as allocentric (i.e., external from the body) reference frame (RF) for the extrapersonal space categorization, the motion potentialities intrinsic to it influence our judgments. However, it is not known whether the same happens when the other body is only a task-irrelevant presence within the scene.

In order to answer this question, we investigated whether egocentric spatial categorization (a judgment about the subjective closeness of a target respect to our own body) could be influenced by the presence of another human body in the scene (Exp. 1), and the relative role of human-like morphology and action opportunities (Exp. 2).

## Experiment 1

We aimed at investigating whether the co-presence of another individual in the extrapersonal space, influences egocentric spatial categorization, as it does in the peripersonal space. The individual in the scene could be processed as a like-me human being, with our same action potentialities (i.e., able to move towards the target) and we could implicitly filter our space categorization considering such motor abilities.

### Materials and Methods

**Figure 1:**
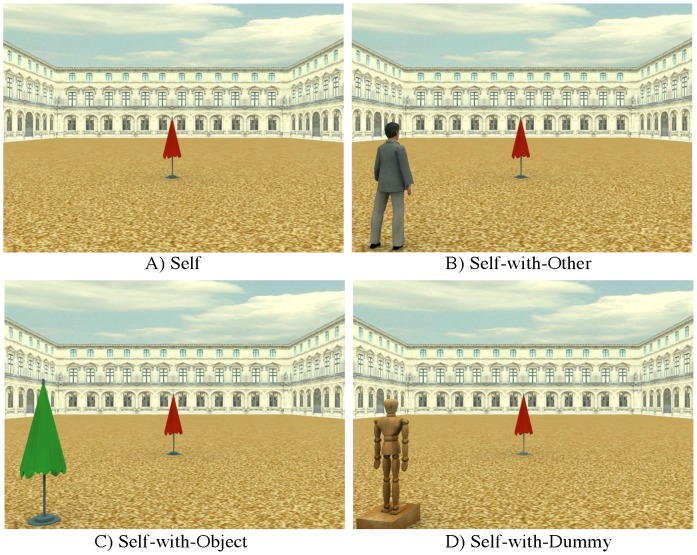
Exemplar stimuli used in the two experiments. A) Self; B) Self-with-Other; C) Self-with-Object; D) Self-with-Dummy.

Fifteen healthy participants took part in this experiment (10 females, mean age 25 years, range 20–29). Stimuli included 3D virtual scenes created by means of a 3D modelling software (3D Studio Max 4.2, Autodesk, Discreet). The scene was a 3D environment, representing a square arena in a park with a three-winged palace. In a first set of stimuli (Fig. 1A), within the above described environment, a red beach umbrella could be located at 27 different distances (from 2 m to 54 m in steps of 2 m) along a central vector aligned to a central camera (height: 160 cm) representing the participant's egocentric perspective (Self condition). In a second set of stimuli (Fig. 1B) an avatar (height: 177 cm) could be located 45° either clockwise or contraclockwise to the central camera, facing the red beach umbrella (Self-with-Other condition). A third set of stimuli (Fig. 1C) was identical to the second one, except for the presence of a green beach umbrella (height: 192 cm) instead of the avatar (Self-with-Object condition). We administered the stimuli through the limits method [29].

This is a method for measuring perceptive thresholds in which the investigated perceptual characteristic is presented with increasing or decreasing values in separate and randomized ascending or descending series, respectively. Here, each experimental series started with a white fixation cross (1.5°×1.5°) on a black background (2500 ms) and consisted of 27 potential trials in which the red beach umbrella was located at 27 different distances from the central camera. Each trial lasted 2500 ms and was followed by a white fixation cross on a black background for 2500 ms. Subjects were asked to judge whether the red beach umbrella was “Near” or “Far” from them, i.e. from their egocentric perspective corresponding to the central camera, by pressing two different buttons. Response buttons were arranged horizontally on a button box and counterbalanced between subjects. The “Near”/“Far” judgments were purely subjective and had to be expressed within stimulus duration (i.e., 2500 ms). In ascending series, the red umbrella was progressively moved away from the central camera until participants provided three consecutive “Far” judgments. In descending series, the red umbrella was progressively moved closer to the RF until participants provided three consecutive “Near” judgments. This was done to ensure judgements consistency. Then the following series started.

The point where participant expressed a transition from “Far” to “Near” (descending series) and from “Near” to “Far” (ascending series) was called *Judgment's Transition Threshold* (JTT). JTT, expressed in meters, was calculated in each series for each subject. Series were then averaged together to obtain a JTT final mean referring to the different conditions. Higher JTT values show a categorization of space as “Near” at longer target distance compared to JTT lower values. Each series was repeated 4 times for each condition. Each subject was thus submitted to 24 randomized experimental series (3 conditions: Self, Self-with-Other, Self-with-Object x 8 series type: 4 ascending, 4 descending). Before starting the experiment, we instructed the subjects about the task to perform and showed them the three sets of stimuli.

Stimuli were presented at full screen on a 17′ computer display placed 57 cm from the subject. The presentation of the stimuli and the recording of the participant's responses were controlled by a custom software (developed by Gaspare Galati at the Department of Psychology, Sapienza Università di Roma, Italy), implemented in MATLAB (the MathWorks Inc., Natick, MA, USA) using Cogent Graphics (developed by John Romaya at the LON, Wellcome Department of Imaging Neuroscience, UCL, London UK).

### Ethics Statement

Participants provided written informed consent before the beginning of the experiment, which was conducted in accordance with the ethical standards of the 1964 Declaration of Helsinki.

### Results

**Figure 2:**
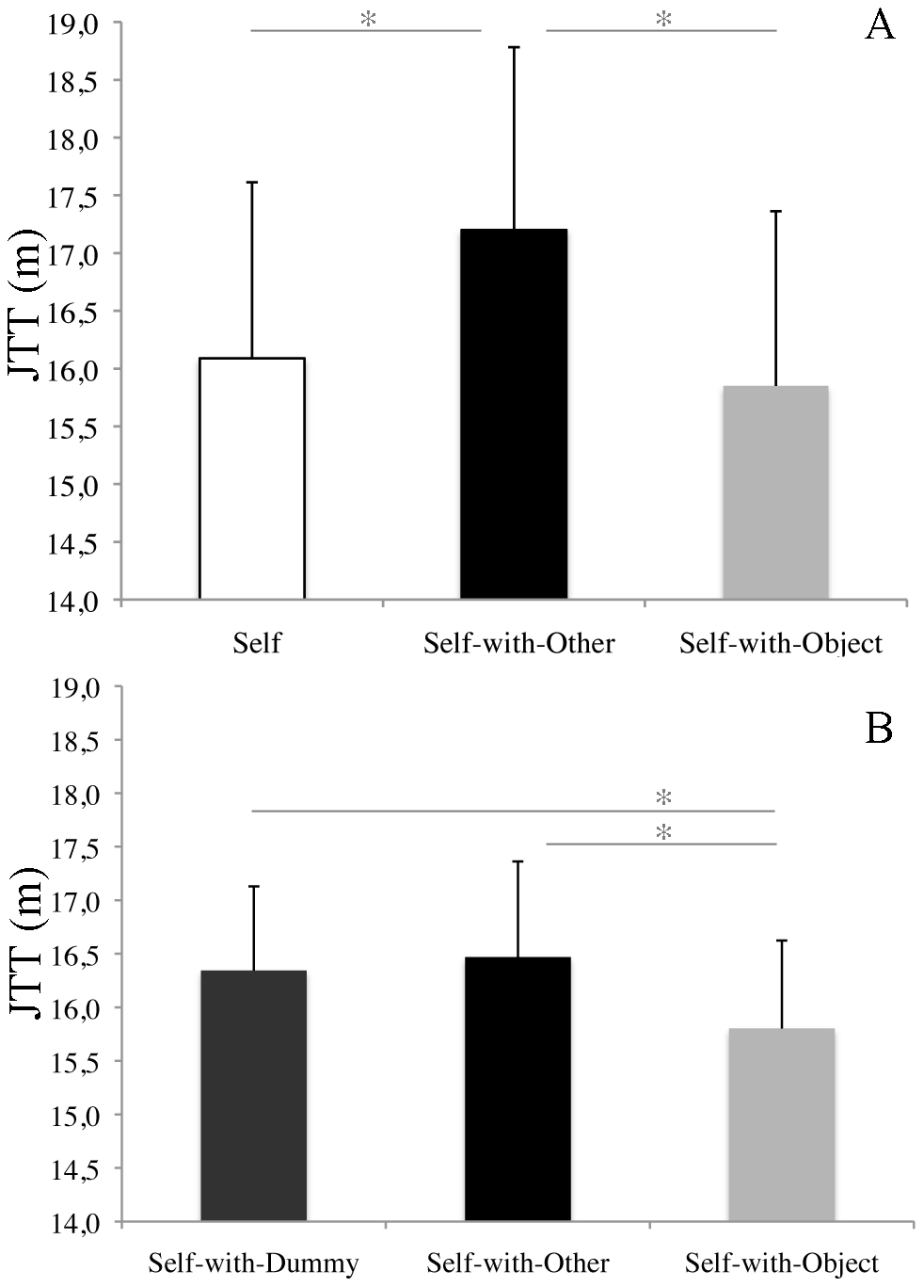
Mean Judgment Transition Thresholds (JTTs). Mean JTTs in the Experiment 1 (A) and the Experiment 2 (B). The error bars indicate the standard errors.

The repeated measures ANOVA comparing JTT in the three experimental conditions (Self, Self-with-Other, Self-with-Object) showed that egocentric spatial categorization significantly changes as a function of the element in the visual scene (F_(2,28)_ = 14.89, p<0.001, η^2^ = 0.52) (Fig. 2A). Post-hoc tests (Newman Keuls) showed higher JTT in the Self-with-Other condition (JTT = 17.2 m, SD = 5.92 m) as compared to Self-with-Object (JTT = 15.85, SD = 5.66 m, p<0.001) and Self (JTT = 16.08 m, SD = 5.7 m, p<0.001) conditions.

Results suggest that the presence of another human body induces a shift in the transition threshold, making far objects appearing closer than they actually are.

## Experiment 2

In the first experiment we found that a human body located in the visual scene impacted on the Judgment Transition Threshold. Nevertheless, previous studies have shown that human-like shapes (e.g. robot), as well as human bodies, impact on our perception of actions and environment around us [30]–[32]. For instance, in the domain of action perception, Oberman and colleagues [33] showed that mu-rhythm, a functional measure of the sensory-motor system, is reduced when participants view action performed both by human and human-like bodies. This finding receives additional support from functional magnetic resonance studies in both humans [30] and monkeys [31].

In a second study we investigated whether the impact of the avatar on Judgment Transition Threshold is selectively tuned to human bodies or a human-like shape, without movement potentialities (a wooden dummy), is enough for the effect to occur.

### Materials and Methods

Twenty-four healthy participants took part in this experiment (20 females, mean age 21,2 years, range 20-23). Twenty-three were right-handed. All participants had normal or corrected-to-normal visual acuity and were naïve as to the purposes of the experiment. Experimental stimuli were the same as in the previous experiment except for the Self condition which was replaced by a Self-with-Dummy condition (Fig. 1D). In this condition a wooden dummy (height: 177 cm) was located 45°, either clockwise or contraclockwise, to the central camera, facing the red beach umbrella. Experimental task and procedure were the same as in the previous experiment.

### Ethics Statement

Participants provided written informed consent before the beginning of the experiment, which was approved by the Ethics Committee of the “G. d′Annunzio” University, Chieti, and was conducted in accordance with the ethical standards of the 1964 Declaration of Helsinki.

### Results

The repeated measures ANOVA comparing JTT in the three experimental conditions (Self-with-Dummy, Self-with-Other, Self-with-Object) showed that egocentric spatial categorization significantly changes as a function of the element in the scene (F = _(2,46)_ = 3.64, p<0.05, η^2^ = 0.14) (Fig. 2B). Post-hoc tests (Newman Keuls) showed higher JTT in Self-with-Other (JTT = 16.46 m, SD = 4.37 m, p<0.05) and Self-with-Dummy conditions (JTT = 16.34 m, SD = 3.85 m, p<0.05) as compared to Self-with-Object condition (JTT = 15.80 m, SD = 4.02 m).

Results suggest that a human-like shape (i.e. a wooden dummy), as well as a human body, induces a shift in the transition threshold, making far objects appearing closer than they actually are.

## Discussion

The aim of the present study was to investigate whether a human body located in the extrapersonal space could influence the self-centred, egocentric space categorization. We showed, in a virtual environment, that both a human body (Exp. 1) and a human-like shape (i.e. a wooden dummy) (Exp. 2) enlarge the space categorized as near.

In the peripersonal space it has been shown that the presence of others is able to modulate our predisposition to act towards a graspable object [16], or simply to see someone reaching an object with a tool extends our perception of the peripersonal space [12], [18]. Our results extend the evidence of the other's influence in spatial perception from the peripersonal space to the extrapersonal space, a space that we commonly share with other people. In fact, as observing someone using a tool to reach a target can extend our peripersonal space, seeing a human body potentially able to cover a distance, can enlarge the portion of space judged as “near space” and consequently reduce our space perception. Another human body could be processed with intrinsic action potentialities tailored in response to the space context, which means linked with the arms in the peripersonal space, and linked with the legs in the extrapersonal space. In accordance with this, we have recently found that the portion of extrapersonal space judged as near is greater when using another body with available movement potentialities as allocentric reference frame [28].

It has been previously demonstrated that our explicit perception of the extrapersonal space varies not only with the optical factors and oculo-motor variables, but also as a function of the energetic costs associated with performing an intended action [4]. According to Proffitt [4], [5], indeed, visual perception of the physical world is not simply a function of optically specified objective features of the environment, but is constrained by the perceiver's capacity to act on that given space, at a given time. For instance, Bhalla and Proffitt [1] employed a task of perceptual estimate about a steep hill when people were encumbered by wearing a heavy backpack, fatigued after a long run, of low physical fitness, or elderly or in poor health. They found that the slant judgements were increased by the reduction in physiological potential brought about by all of the above experimental manipulations.

Interestingly, the present results showed that the other's body would determine a reduction of the perceived space even when the perceptual judgement is self-centred and the other's human body is only an element incidentally present in the environment.

Noteworthy, the human body is able to reduce the perceived space also when it is a static wooden dummy. Structural and morphological shapes resembling human bodies are therefore sufficient conditions for the effect to occur. During the egocentric spatial categorization an implicit, low-level analysis of the body shape seems thus to be at play. The effect might be mediated by the extrastriate body area (EBA) within the inferior temporal sulcus [34] which is activated by images of humans (both moving and static) presented in a variety of formats (e.g. photographs, line drawings, silhouettes) relative to perceptually-matched control objects, as well as during the vision of human biological movement [35]. Thus, given the ability of our brain to extract motor information even from static pictures, we arise the possibility that a wooden dummy might be sufficient to trigger the biological motion's representation that impacts on our space perception.

Conversely, when explicitly using the dummy as allocentric RF for the spatial categorization, we failed to replicate the effect [28]. While the egocentric representations are readily encoded in the dorsal stream as sensorimotor representations, allocentric ones are mainly encoded in the ventral stream as perceptual representations [36]-[40]. So, when the RF is allocentric, categorizing space might imply a finer processing of the RF's characteristics and consequently the “living” appearance of the human structure could play a greater role.

It could be argued that our study suffers from subjective size and distance estimation because of the lack of fine depth cues (which are available only in the real setting or in an immersive virtual reality setup), as well as from a high size familiarity of the human body respect to the control object (the beach umbrella). This is, however, a problem potentially without solution because all objects in the world can have different sizes and are less familiar than the human body. To this regard, it is worth noticing that the present study did not aim at extracting absolute values of distance estimation, but rather at investigating the intrinsically subjective experience of the surrounding extrapersonal space. To this aim, we chose a dichotomic categorization task based on verbal labels (rather than continuous metric evaluations). This task employs categorical spatial representations, which serve perceptual functions such as registering positions in both egocentric and allocentric reference frames [41]. We opted for the “Near/Far” judgement because of its ecological value. When we have to decide to cross a street, or walking instead of taking a bus, we judge very rarely in metrical terms, but we implicitly judge if something is “Near” or “Far” (likely taking into account our possibility to act [5]). Despite this, to rule out the presence of basic biases due to our 3D virtual setup, we asked to a different sample of subjects to perform a one-shot estimation in meters of a 16 meters-long distance in the virtual scene (corresponding to our average judgment transition threshold in the Self condition, see Exp. 1). We obtained an average metric estimation of 8.20 meters, which is in accordance with data showing that distances in virtual environments are processed almost twice compressed than distances in real space (e.g., [42], [43]).

In conclusion, when sharing the extrapersonal space with other bodies, a wider portion of space appears as near and therefore “accessible”. This might represent an evolutionary advantage in the struggle to survive in presence of potential competitors within the environment, although further studies are needed in both virtual and ecological reality to ascertain our speculation.
